# Impact of COVID‐19 on student attainment and pedagogical needs when undertaking independent scientific research

**DOI:** 10.1111/ahe.12842

**Published:** 2022-07-23

**Authors:** Jennie N. Jeyapalan, Victoria. James, David S. Gardner, Jennifer H. Lothion‐Roy, Nigel P. Mongan, Catrin Sian Rutland

**Affiliations:** ^1^ School of Veterinary Medicine and Science, Medical Faculty University of Nottingham Nottingham UK

**Keywords:** assessment, COVID‐19, research, veterinary

## Abstract

Research is often an essential component of completing a veterinary medicine degree, with universities worldwide aiming to teach students a variety of techniques and general research comprehension and skills. As universities worldwide navigated the COVID‐19 pandemic, it was often necessary to move towards distance learning, this was employed for the research module at The University of Nottingham, School of Veterinary Medicine and Science. Following completion of their independent research project, each student cohort was sent a student evaluation of the module questionnaire and quantitative and qualitative analysis was undertaken. In addition, assessment outcomes based on dissertation grade, supervisor grade and overall module score were analysed quantitatively. This was conducted on both the individual cohorts and between the pre‐ and peri‐pandemic groups, ranging from 2017–2018 through to 2021–2022 cohorts. The students received increased dissertation and supervisor grades (by nearly 6%) during the 2021–2022 peri‐pandemic cohort, when compared to the pre‐pandemic cohorts, but did differ significantly compared to the 2020–2021 cohort. The pre‐ and peri‐pandemic Likert‐scale ratings for module organisation and assessment criteria were similar, workload management and the ability to explore concepts and ideas was reduced in the peri‐pandemic cohorts, whereas the accessibility to resources was increased in the peri‐pandemic students compared to those taught prior to the pandemic. Student feedback can provide essential information when designing and managing research projects and when compared to assessment grades it can help us understand attainment, essential information when providing a quality university level education whilst supporting student welfare following the COVID‐19 pandemic.

## 1 INTRODUCTION

University education was affected worldwide as COVID‐19 turned into a pandemic affecting, only five countries have not reported active cases to date (for latest World Health Organization data see [World Health Organization, 2022]). Universities in the United Kingdom commenced distance learning, gradually moving to more blended distance learning alongside face‐to‐face teaching. Veterinary medicine courses were able to operate blended learning with enhanced health and safety procedures within a few months. Following the onset of the COVID‐19 pandemic, the School of Veterinary Medicine and Science, University of Nottingham (SVMS) transitioned to distance learning, with all undergraduate learners conducting their teaching, learning, pastoral care, assessment and research activities remotely. Four months later SVMS started hybrid/blended teaching, with both face‐to‐face and distance learning continuing into 2022 (two academic cohorts).

Much has been written about student experience throughout the pandemic, and about in‐person learning versus blended and distance pedagogical approaches (Brombacher‐Steiert et al., 2021; Ellaway et al., 2003; Little et al., 2018; Routh et al., 2021). Despite this, few studies have looked at performance outcomes, and very few have used mixed methods to determine both performance outcomes and student experience and compared pre‐pandemic and peri‐pandemic outcomes. Blended learning has generally produced relatively good student satisfaction levels, although this is difficult to assess given the differing blended learning models, disciplines and circumstances under which they were applied. In examples from biology, biochemistry and genetics, no differences in examination outcomes were observed between in person, blended or distance learners (Leatherman & Cleveland, 2020; Stockwell et al., 2015; Tahir et al., 2022). Whilst these traditionally practical and lectures‐based courses were generally well received by students and exam performance was similar between the groups. Independent research projects are very different to these types of courses and from many of the other elements of traditional university degrees.

It has been suggested that students were able to adapt to new distance learning studies during the pandemic, due to use of technologies in their general life and previous university studies (Limniou et al., 2021). In anatomy, use of both traditional methods and newer modern techniques such as 3D printing and virtual reality showed statistical similar results in student attainment (Iwanaga et al., 2021).

Independent research projects are common elements of undergraduate degrees worldwide, serving to guide students through research whilst also encouraging future research careers. At SVMS, every veterinary undergraduate student undertakes a compulsory individual research project lasting 8–12 weeks. The Bachelor of Veterinary Medicine and Bachelor of Veterinary Surgery (BVMBVS) degree is achieved following five years of study, students additionally benefit from having an integrated Bachelor of Veterinary Medical Sciences (BVMedSci) degree, which is achieved at the end of year three, incorporating the research project. In the United Kingdom, degrees are graded 0%–100%, classified as 40%–49% Third‐Class, 50%–59% Lower Second‐Class, 60%–69% Upper Second‐Class and 70%–100% is classified as a First‐Class degree. The research project is a high credit module and plays an important role in overall degree classification.

The research project topics are varied at SVMS and can be conducted within any theme allied to veterinary medicine and science, including anatomy, histology and embryology. It is not a traditional didactic course as the students work one‐to‐one with an academic to complete a unique research project. Traditionally this may have included laboratory or fieldwork elements, or other types of research including surveys, data analysis, literature reviews or analysis of results collated by academic supervisors.

Naturally, gathering and analysing feedback about research teaching, learning and assessment, and understanding student assessment outcomes is essential when delivering quality teaching. The aim of this paper was to investigate whether student feedback and achievements (in terms of assessment grades) differed in three pre‐pandemic cohorts compared to two peri‐pandemic cohorts. A mixed methods approach was taken to understand student experience and assessment performance.

## 2 MATERIALS AND METHODS

### 2.1 Cohort information

This research project was reviewed and approved by the School's Committee for Animal Research and Ethics (CARE), School of Veterinary Medicine and Science, University of Nottingham (ethics number 3555220223). The independent research project is a compulsory module undertaken by every undergraduate student within SVMS. Each student conducts research with a designated academic supervisor and assessment consists of a 6000 word dissertation (90% of module grade) and a supervisor grade (10% of module grade).

Five individual cohorts were investigated: 2017–2018 *n* = 135, 2018–2019 *n* = 148, 2019–2020 *n* = 153, 2020–2021 *n* = 161, 2021–2022 *n* = 150. These individual cohorts were additionally grouped into (A) a pre‐pandemic group consisting of cohorts 2017–2018, 2018–2019 and 2019–2020, *n* = 436 and (B) a peri‐pandemic group, which included cohorts 2020–2021 and 2021–2022, *n* = 311. 100% of the undergraduate students were enrolled onto the research module each year. The length of the research projects module was 12 weeks in 2017–2018, 10 weeks in 2018–2019 and 2019–2020, and 8 weeks in 2020–2021 and 2021–2022, equating to 30 credits. In 2017–2018, the overall module grade included a viva component, which was discontinued thereafter; therefore, overall module grades were only compared for the four latter years. With this exception, the marking criteria for both the dissertation and supervisor grades elements remained the same throughout the cohorts.

### 2.2 Evaluation of student assessment outcomes

Assessment grades based on (1) the dissertation and (2) the supervisors' assessment of the students' progress throughout the project and (3) the overall module grade, were analysed. Both assessments had a marking scheme and were based on a 0–100 scale. Dissertations were assessed by two examiners, with a third employed where grades differed by more than 10% and for dissertations not achieving the 50% pass threshold grade. Kruskal‐Wallis with Dunn's multiple comparisons test was used to compare the five individual cohorts and the Mann–Whitney U test to compare the pre‐ and peri‐pandemic groups. *p* < 0.05 was considered statistically significant.

### 2.3 Evaluation of student feedback

Following the completion of each annual research projects module, all students were invited to evaluate the module anonymously using a 1–5 Likert‐scale scoring system (Jamieson, 2004). The respondents were additionally provided open text comment feedback opportunities.

Statements:

1. The module helped me to explore ideas/concepts/topics in depth.
2. The module was well organised.
3. The module resources were easily accessible.
4. The criteria for assessments were clear.
5. The module workload was reasonable/manageable.
6. The online provision during COVID‐19 was satisfactory.

The Likert scale ranged from 1–5: 1 = Strongly disagree, 2 = Disagree, 3 = Neutral, 4 = Agree, 5 = Strongly Agree. Statement 6 was only provided to the 2021–2022 cohort. Quantitative analysis was undertaken on the student ratings for each statement. The Median (min‐max) was calculated for each individual cohort and the pre‐ and peri‐pandemic groups. In addition, qualitative with semi‐quantitative analysis was undertaken on the open comments via classification of each concept/comment.

## 3 RESULTS

In the pre‐pandemic cohorts, the projects undertaken consisted of literature reviews (narrative or systematic), meta‐analysis dissertations and analysis based on surveys. Students were also able to analyse data from laboratory work, clinics or fieldwork (undertaken by the supervisor or the student), which included digital slide scans, CT/MRI/X‐rays/other imagining techniques, biochemistry and epidemiological data, in vitro and in vivo outcomes, metabolomic/genome/bioinformatics/sequence/structure data and databases, and data from clinics such as ECGs, blood pressure and pharmacological data. In addition, the students were able to develop tools and models. In the peri‐pandemic cohorts, the only difference in project type observed was that students were not personally able to enter laboratories or undertake fieldwork, but they were still able to analyse and present the results from laboratory and fieldwork.

Examples of projects included literature reviews and meta‐analysis of anatomical structures (remained unchanged pre‐ to peri‐pandemic). Histological scoring and analysis of protein expression in tissues, where pre‐pandemic the students would have undertaken immunohistochemistry but peri‐pandemic the supervisor conducted the immunohistochemistry and all students undertook H‐scoring. Data projects such as performance levels, surgery recovery rates, epidemiological studies and analysis of clinical images (e.g. computed tomography, X‐ray, magnetic resonance imaging) remained the same pre‐ and peri‐pandemic. Laboratory projects, which may have involved doing polymerase chain reaction were undertaken peri‐pandemic using bioinformatics or analysis of supervisors’ laboratory results. Prior to the pandemic, around 30% of the cohort undertook laboratory or field based projects.

**FIGURE 1:**
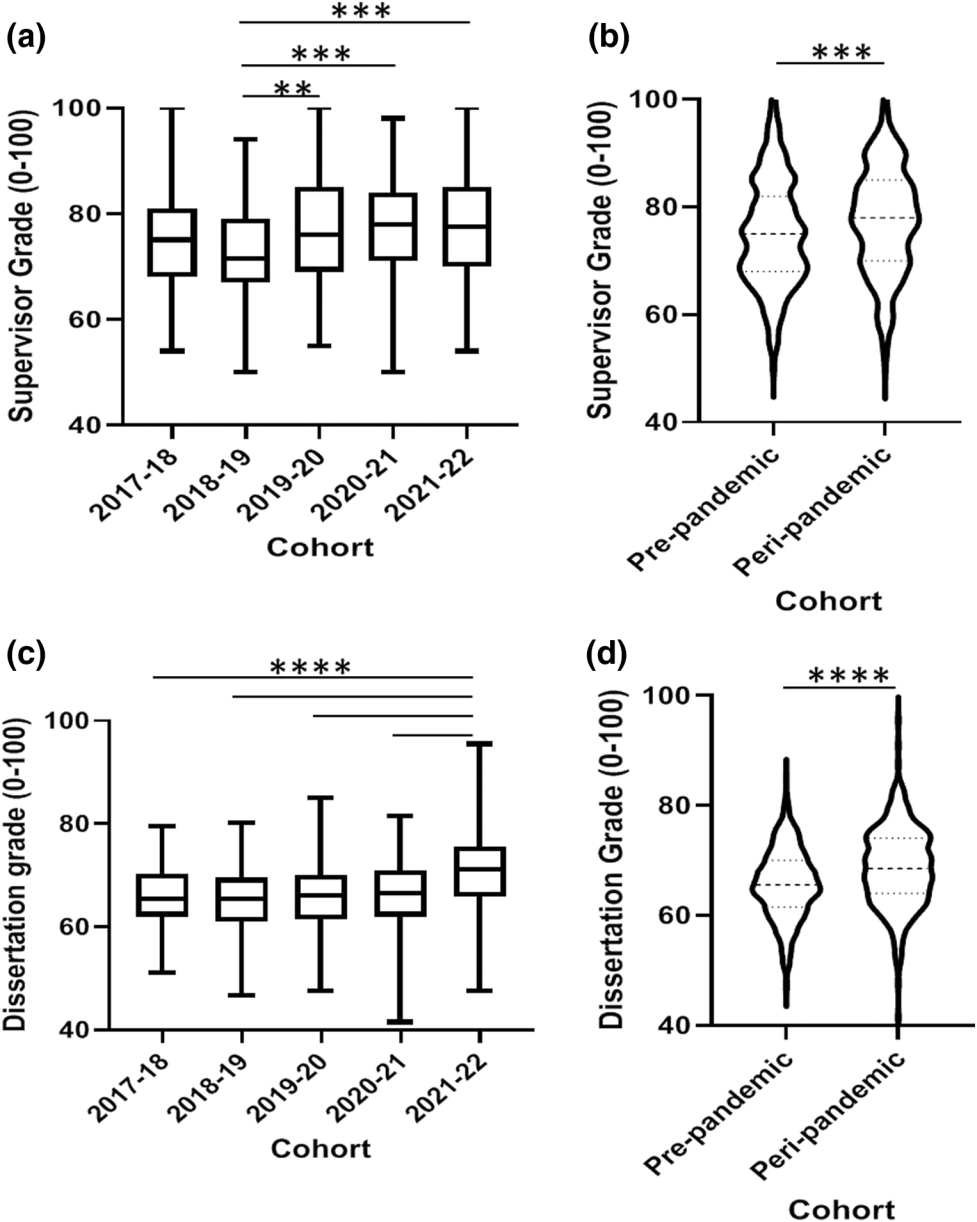
Dissertation and supervisor grade outcomes. Dissertation grade by (a) individual cohort and (b) pre‐ and peri‐pandemic. Supervisor grade by (a) individual cohort and (b) per‐ and peri‐pandemic. (a and c) **p ≤ 0.01, ***p ≤ 0.001, ****p < 0.0001 Kruskal‐Wallis with Dunn's multiple comparisons test. *N* = 135 2017–2018, *n* = 148 2018–2019, *n* = 152 2019–2020, *n* = 161 2020–2021, *n* = 150 2021–2022. (b and d) ***p = 0.002 Mann, ****p < 0.0001 Mann–Whitney U test. *N* = 436 pre‐pandemic, *n* = 311 peri‐pandemic

**FIGURE 2:**
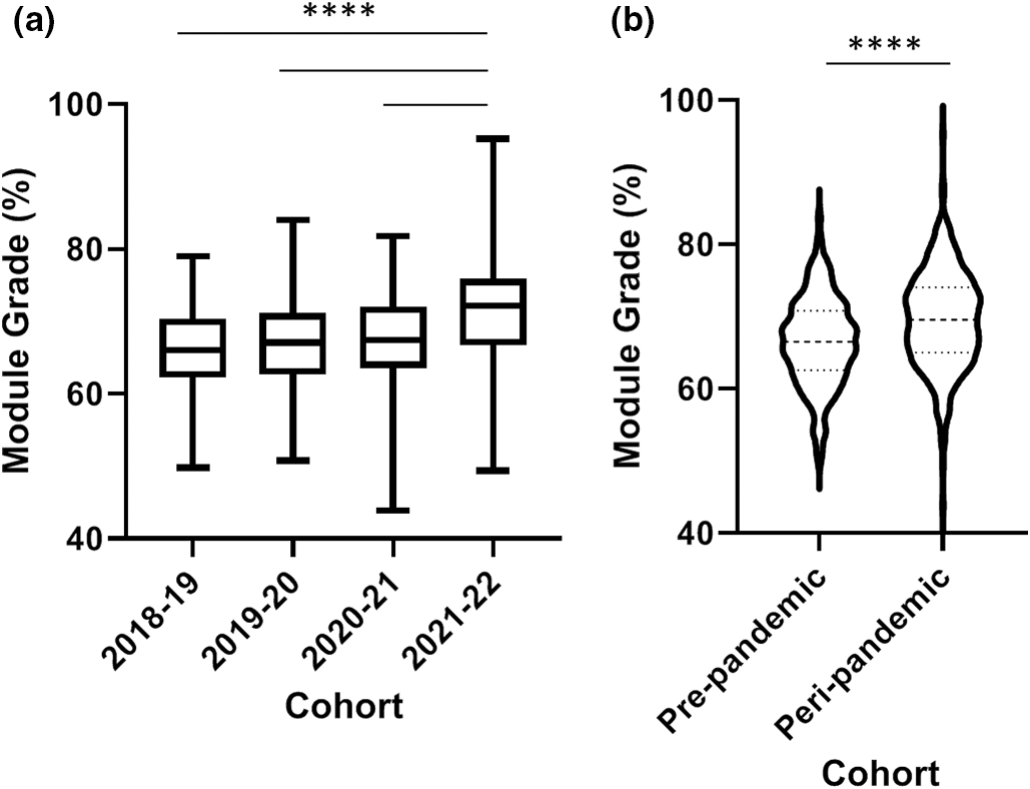
Overall module grade. (a) Grade by individual cohort ****p < 0.0001 Kruskal‐Wallis with Dunn's multiple comparisons test. N = 148 2018–2019, *n* = 152 2019–2020, *n* = 161 2020–2021, *n* = 150 2021–2022. (b) Pre‐ and peri‐pandemic grades **p < 0.0001 Mann–Whitney U test. *N* = 301 pre‐pandemic, *n* = 311 peri‐pandemic. 2017–2018 not included as it included a viva grade

A significant increase of 6% was observed in dissertation grade in the 2021–2022 cohort compared to each of the previous four cohorts (*p* < 0.0001; Figure 1a). The 2020–2021 pandemic cohort did not exhibit significant grade differences compared to the pre‐pandemic cohorts (*p* > 0.05). Overall this resulted in a significant increase in peri‐pandemic dissertation grades compared to pre‐pandemic (*p* < 0.0001; Figure 1b). The 2018–2019 cohort (pre‐pandemic) exhibited the lowest supervisor grades of all five cohorts and was significant lower than the 2019–2020, 2020–2021 and 2021–2022 cohorts (*p* < 0.01, 0.001 and 0.001, respectively), but not significantly different to 2017–2018 (*p* > 0.05; Figure 1c). Supervisor grades were increased by 3% in the peri‐pandemic cohorts when compared to the pre‐pandemic cohorts (*p* < 0.001; Figure 1d). In terms of overall module grade (weighted more heavily towards the dissertation element), a 5.7% increase was observed in the 2021–2022 cohort, which was significantly higher than 2018–2019, 2019–2020 and 2020–2021 (*p* < 0.0001; Figure 2a). This resulted in an overall peri‐pandemic increase of 2.96% compared to the pre‐pandemic cohorts (*p* < 0.0001; Figure 2b).

**FIGURE 3:**
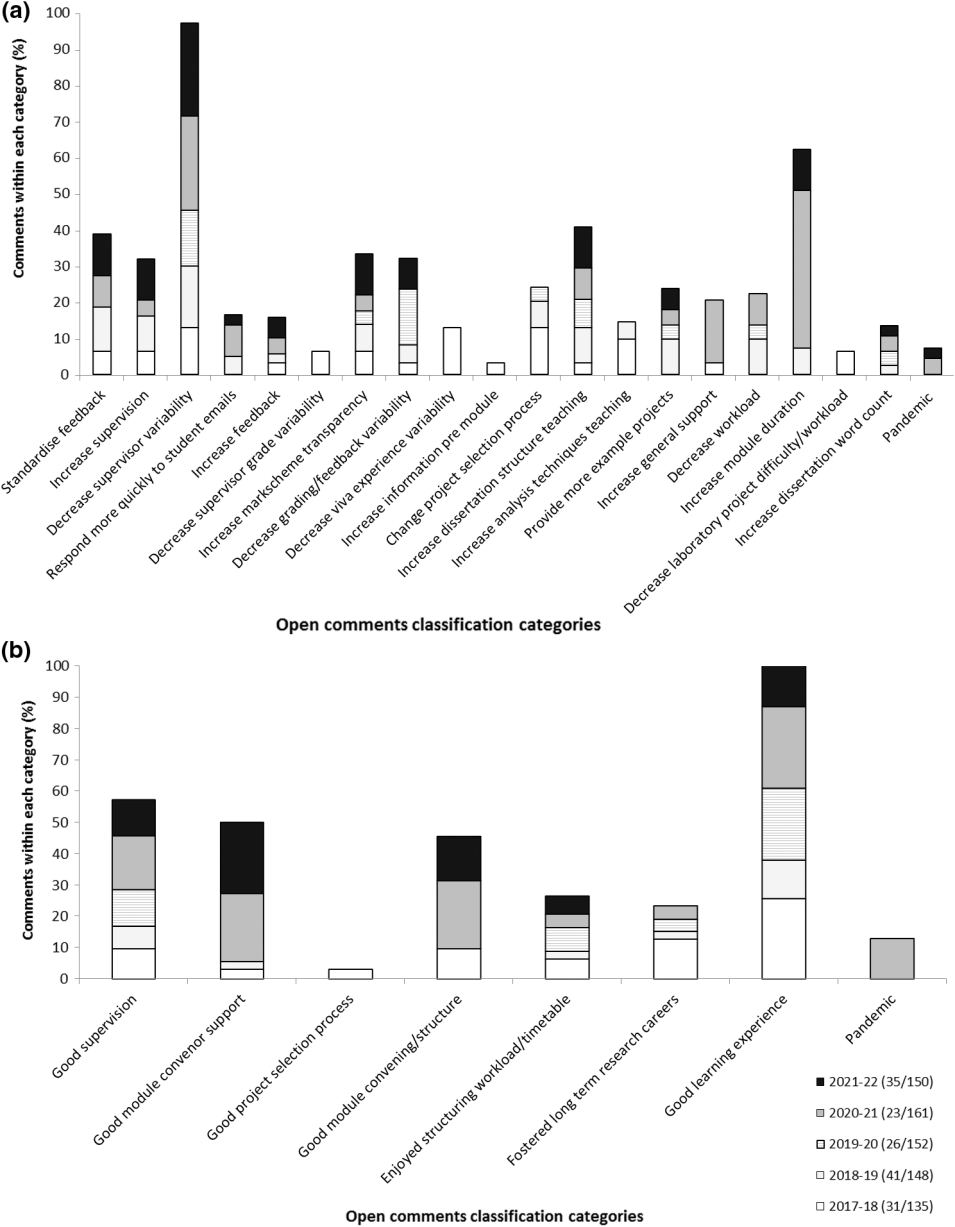
Open comment themes. (a) Areas to improve. (b) Positive experiences feedback

The total number of students responding to the survey per cohort was 31 from 135 (23%) in 2017–2018, 41/148 (27.7%) in 2018–2019, 26/152 (17%) in 2019–2020, 23/161 (14.3%) in 2021–2021 and 35/150 (23.3%) in 2021–2022. The open comments analysis were subdivided into areas to improve, (Figure 3a) and positive feedback/positive experiences (Figure 3b).

The highest number of open comments regarding areas to improve across the five cohorts centred around reducing supervisor variability (for example in relation to teaching styles/contact time/feedback variability from supervisor to student). In addition, some students wanted to increase the duration of the research module. Notably only pre‐pandemic students reported they wanted to increase the amount of information they received prior to the module commencing. They also wanted to change the method of project selection and to increase the amount of teaching on analysis techniques (for example statistics and graphs). The pre‐pandemic cohorts also wanted to decrease laboratory time and decrease viva voce examination variability, neither of which were factors encountered by the peri‐pandemic cohorts as they had no access to laboratories and did not participate in an oral viva voce examination. It was notable that only a small number of students commented on the pandemic as an area of difficulty.

In relation to positive feedback/student experiences, the peri‐pandemic cohorts had a high proportion of students reporting on good supervision, good module convenor support and good module convening structure (Figure 3b). All cohorts included comments relating to good supervision, enjoying being able to structure their own timetable/workload, and stated it was a good learning experience, with some commenting to the effect that it fostered a long term research career, although no comments relating to that were received in 2021–2022. In the 2020–2021 cohort, 10% of students independently stated that the research project was a good experience to have during the pandemic.

The set statements in the student evaluation (Likert scale 1–5) covered teaching, learning and assessments aspects ranging from the capacity to explore ideas or concepts in‐depth, organisation of the module and the ability to complete work and manage workload, the criteria for assessment marking, module resource availability and in the final cohort their satisfaction with the online provision during remote teaching.

**FIGURE 4:**
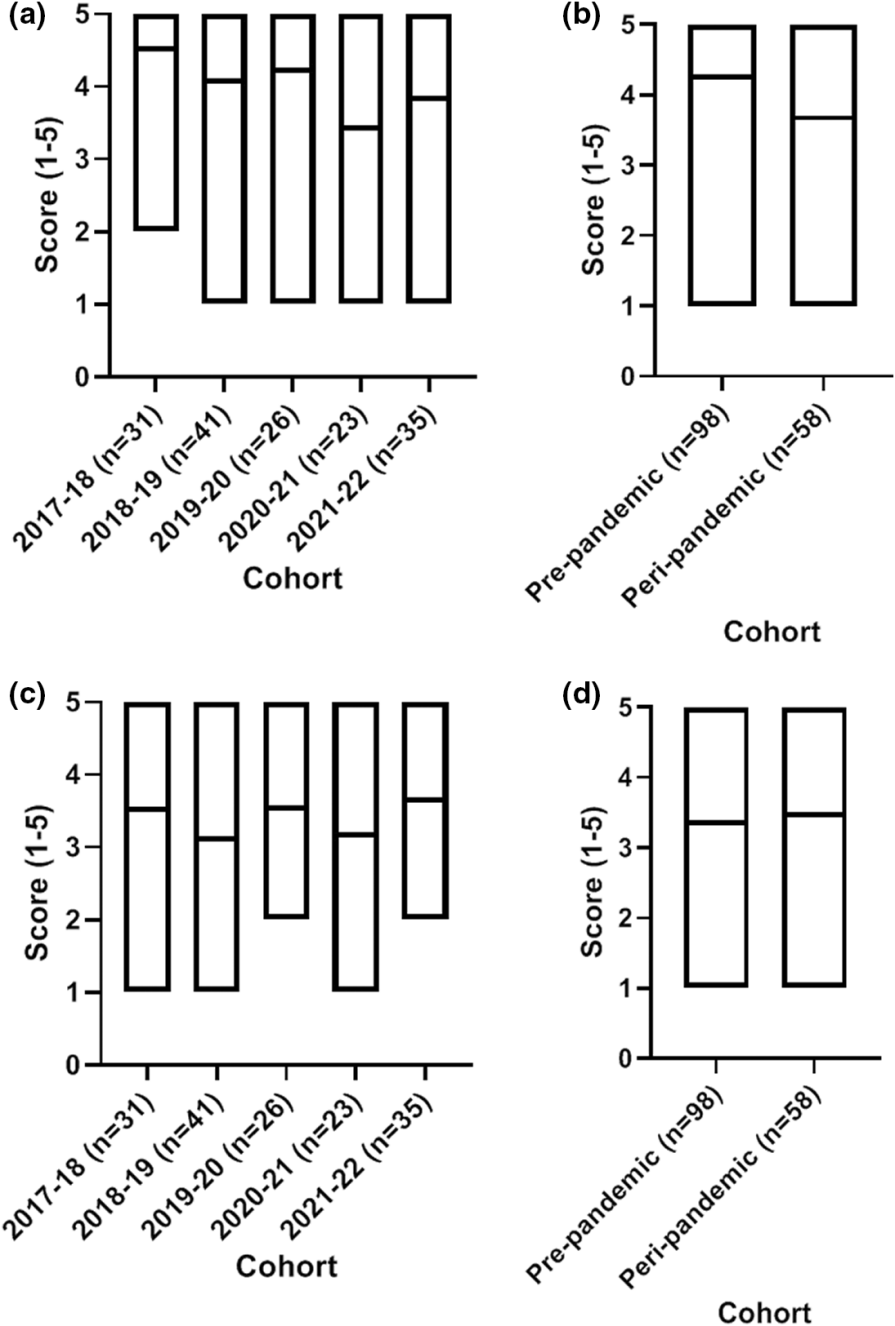
Student experience ratings around module teaching and organisation. The module helped me to explore ideas/concepts/topics in depth by (a) cohort and (b) pre‐ and peri‐pandemic. The module was well organised by (c) cohort and (d) pre‐ and peri‐pandemic. Median (min‐max). 1 = strongly disagree, 2 = disagree, 3 = neutral, 4 = agree, 5 = strongly agree

The statement “the module helped me to explore ideas/concepts/topics in depth” with scores ranging from 3.4–4.5 across the five cohorts, with higher scores pre‐pandemic compared to peri‐pandemic (Figure 4a,b). The statement “the module was well organised” scored 3.2–3.70 across the cohorts, with little variation between the five cohorts or pre‐ and peri‐pandemic (Figure 4c,d).

**FIGURE 5:**
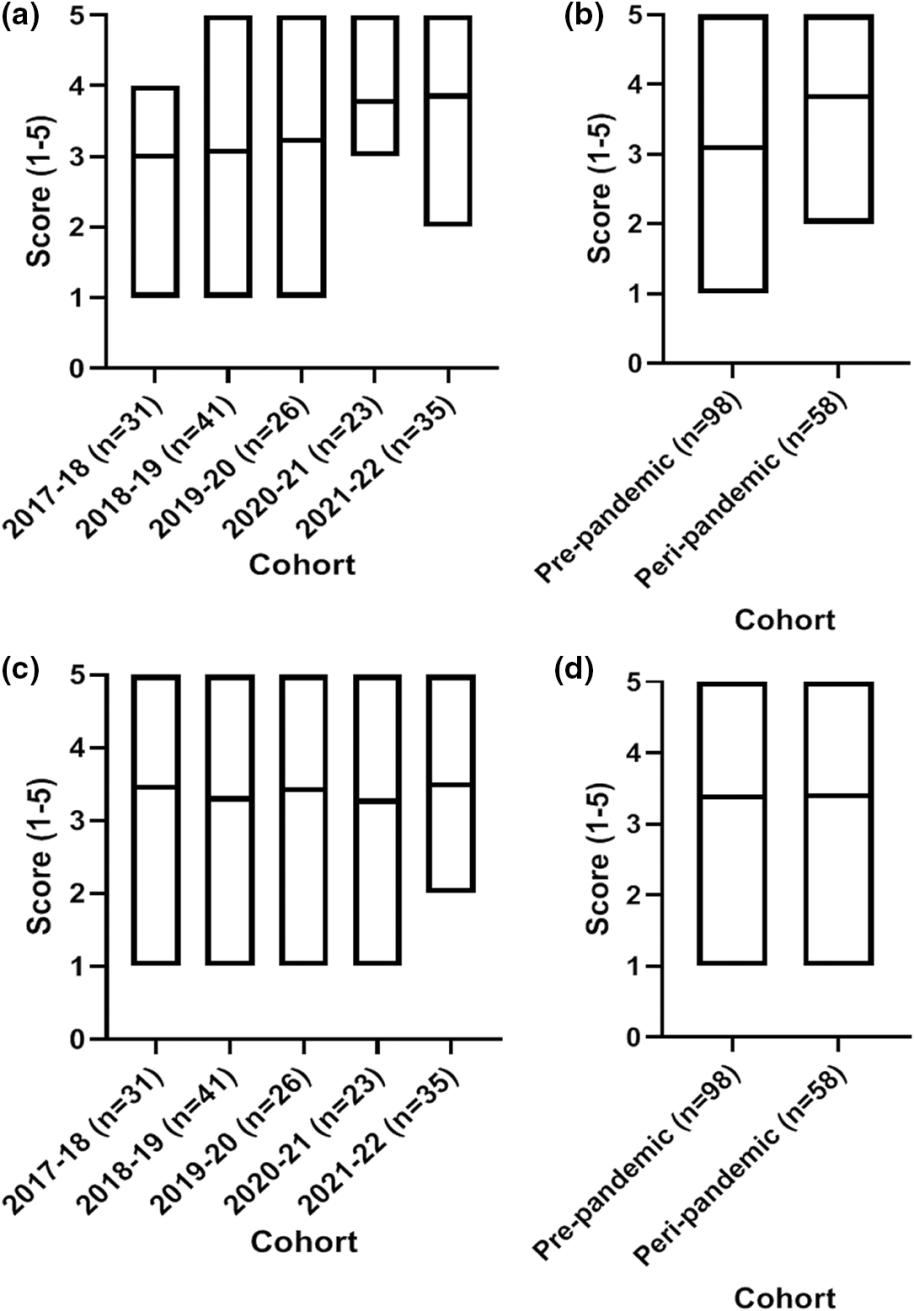
Student experience ratings around resource availability and assessment criteria. The module resources were easily accessible by (a) cohort and (b) pre‐ and peri‐pandemic. The criteria for assessments were clear by (c) cohort and (d) pre‐ and peri‐pandemic. Median (min‐max). 1 = strongly disagree, 2 = disagree, 3 = neutral, 4 = agree, 5 = strongly agree

The student feedback relating to “resources are easily accessible” ranged from 3–3.9, with higher scores in the peri‐pandemic group compared to the pre‐pandemic groups (3.82 vs 3.09; Figure 5a,b). Relating to the statement “the criteria for assessments were clear”, little variation was observed between the ratings given by the individual cohorts (cohorts ranged from 3.3–3.9) or between the pre‐ and peri‐pandemic groups (3.38 vs 3.4; Figure 5c,d).

**FIGURE 6:**
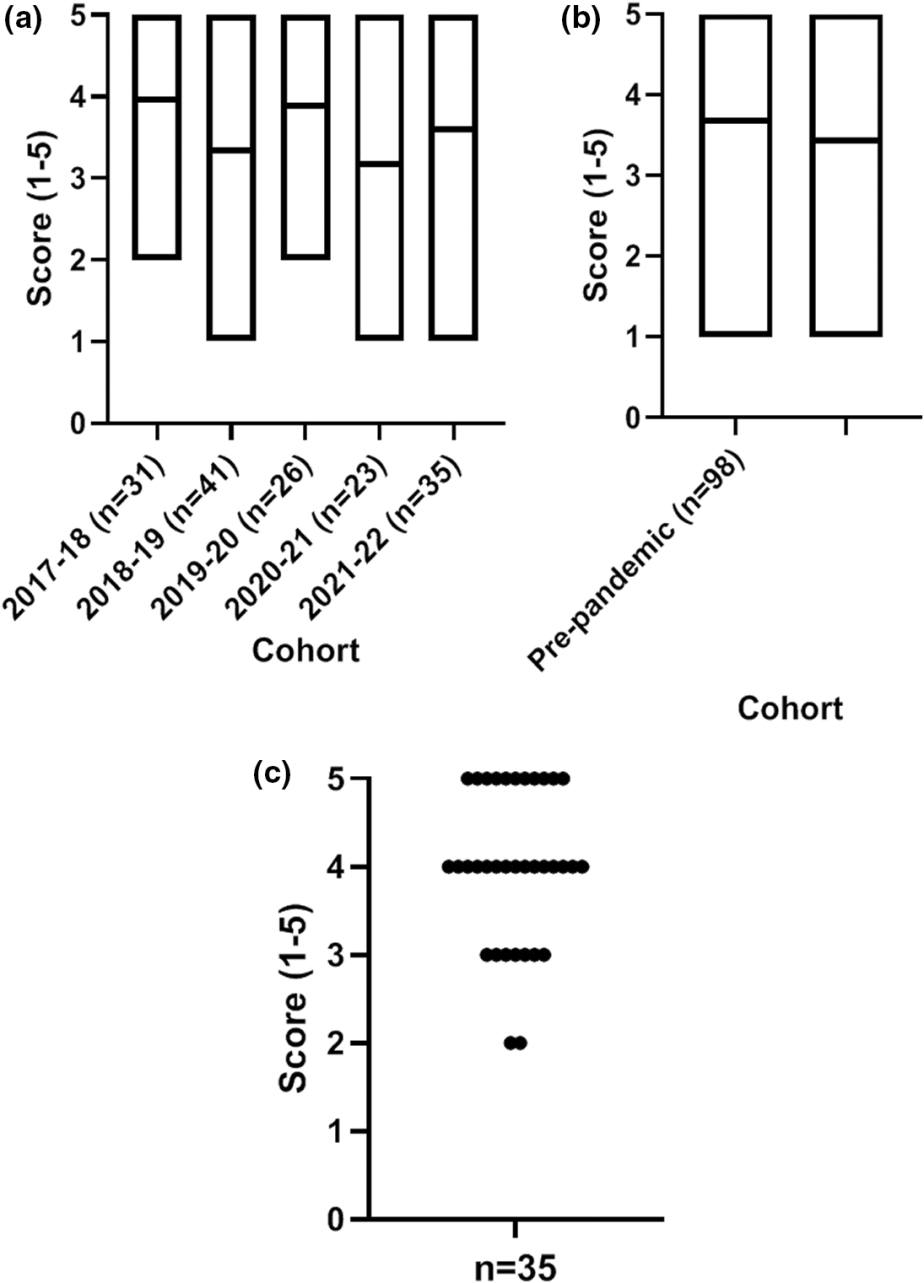
Student experience ratings around workload and online provision. The module workload was reasonable/manageable by (a) cohort and (b) pre‐ and peri‐pandemic. (c) the online provision during COVID‐19 was satisfactory, asked to the 2021–2022 cohort only. Median (min‐max). 1 = strongly disagree, 2 = disagree, 3 = neutral, 4 = agree, 5 = strongly agree

When asked whether the “workload is reasonable/manageable” a greater variation in responses was given, the cohorts ranged from 3.2–4.0. On average the peri‐pandemic response was lower at a score of 3.4 compared to the 3.7 score given by the pre‐pandemic group (Figure 6a,b). In 2021–2022, the standard survey sent to students held an additional statement “online provision during COVID‐19 was satisfactory”. An average score of 4 (agree) was reported, with no students reporting that they strongly disagreed with the statement (Figure 6c).

## 4 DISCUSSION

Prior to the pandemic, SVMS regularly used a variety of online systems to support teaching, learning and pastoral care. Each student has always been provided with a laptop computer for the duration of their degree. During the pandemic, further online resources were developed and implemented to support our learners and help deliver teaching and research. The decision to teach the research projects module using distance learning was made in May 2020 and remained in place for the 2021/2022 cohort. Early decision making was critical to enable staff and students time to design appropriate individual projects, prepare resources, concepts and data, and adapt to the module and project modifications. All these activities to support the learning environment within the research projects, having enthusiastic researchers and highlighting the research relevance to the clinic have been shown to be important for student engagement (Janicke et al., 2020). This also helps explain why the positive student feedback centred around good supervision, module support and generally having a good learning experience.

Competence, autonomy and positive emotions have been shown to be factors important for intrinsic motivation for remote and self‐directed learning (Deci & Ryan, 2000). They have also been linked to resilience (Ryff & Singer, 2003). Together, these factors help reduce stress and promote adaptive coping mechanisms during stressful times (Vansteenkiste & Ryan, 2013; Weinstein & Ryan, 2011). It was, therefore, good to see that the statement “the module helped me to explore ideas/concepts/topics in depth” achieved the highest student rating of all the statements, with average scores reaching 4.5 out of 5. The ability to adapt was possibly enhanced by the advanced knowledge of change provided to the students. The decision to not undertake laboratory of fieldwork was undertaken during a highly variable time, when national lockdowns (government mandated home confinement or reduced interactions with other people) and COVID‐19 procedures were changing rapidly. By making a decision, the students and academic supervisors had time to adapt, thus reducing stress and enabling time for positive emotions to form.

During the COVID‐19 pandemic, resource availability was of concern to universities (UNESCO IESALC, 2020), but students in the present study consistently rated resource availability as higher than the pre‐pandemic cohorts, this was probably due to the increased university and international resources available online. No assessment criteria changes were made throughout the five cohorts, and student ratings remained consistent, but with means ranging from 3.3–3.9, more can be undertaken to make assessment criteria clear for the students. A similar conclusion can be drawn regarding module organisation, although it was noted that student rating increased during the peri‐pandemic period, despite the module not changing appreciably but the students were engaging with the online provision more. It has also been shown that stress over assessment can be reduced by having clear assessment criteria (Bloxham & Boyd, 2007).

The students in the 2020–2021 cohort reported that the workload was less manageable in comparison to the pre‐pandemic cohorts, and it is also important to note that the 2021–2022 cohort ratings were similar to pre‐pandemic ratings. In university level students, fear of contagion, separation from school and importantly academic workload were found to be important stressors (Yang et al., 2021). The 2020–2021 students experienced their research projects just six months into the pandemic, whereas the 2021–2022 cohort were undertaking their research 18 months after the pandemic commenced. The 2020–2021 students were experiencing lockdown situations, vaccines were not yet available, travel was heavily restricted, and uncertainty and instability possibly played a larger part in their day to day lives. A key factor was potentially illness from COVID‐19 itself, or from stress related illness. To try and mitigate this, all students were given “extenuating circumstances” support by the university and additional dissertation writing time, and any students were able to interrupt their studies for a year or more, although most chose not to. Factors such as finances, living away from their family, managing intimate relationships and even coping with caring roles are all factors, which can become additional stress factors (Hurst et al., 2013). The majority of students at SVMS live in accommodation near to the university, with their family homes spread across the United Kingdom or abroad. The peri‐pandemic research project students were able to live in their family home if they wanted to, and potentially this would also reduce financial pressures (a concern during the pandemic [UNESCO IESALC, 2020)], whilst potentially getting emotional support from their family. Although these could be potential positive factors, it has also been shown that distance learning alters student and teacher communication patterns, with an increased level of isolation and independence for the students(Hurst et al., 2013), therefore, in itself, this method of teaching and learning is a stress factor. This stress factor may have been balanced by the positive factors available to the students undertaking their distance learning research projects such as living at home, reduced financial pressure and reduced isolation from family and friends, for some students, enhancing overall well‐being, thus impacting resilience.

The grades achieved by the students undertaking the research module increased by 3% over the peri‐pandemic two‐year period, compared to the pre‐pandemic period. Both the supervisor assessed element and the dissertation grades increased over this period, but most of the increase was due to 2021–2022 rather than the 2020–2021 period when COVID‐19 was a relatively new experience for everyone. Grade inflation has been reported across many aspects of higher education. With instructors potentially compensating for unforeseen negative circumstances (Karadag, 2021). Grade inflation has been a topic of conversation and academic study for many decades now (Astin, 1998; Eiszler, 2002; Popov & Bernhardt, 2013), but it is difficult to assess whether student attainment may have improved due to a reduction in their social life and other factors or due to differences in assessment grading.

Undergraduate independent research projects are especially important within undergraduate degrees (Ávila & Rodríguez‐Restrepo, 2014; Bridge et al., 2018; Cehrs et al., 2020), highlighting the importance of research in the veterinary profession and beyond. The present study highlighted how making early decisions on teaching, learning and assessment procedures and advanced planning and implementation, alongside clear assessment criteria, with a supportive learning environment and enforcing digital capabilities, meant the student experience around this vital research interaction was altered for students undertaking their research during the pandemic. Academic performance was enhanced during the pandemic period, with both peri‐pandemic cohorts achieving higher grades compared to the three cohorts prior to COVID‐19. Stress, mental well‐being and resilience have an impact on workload management and perception of workload (Ryff & Singer, 2003; Vansteenkiste & Ryan, 2013; Weinstein & Ryan, 2011), it is possible these affected the first cohort of students undertaking their research following the pandemic, as highlighted by their feedback relating to workload perceptions.

Understanding the diverse needs of our students both during and following the pandemic, whilst balancing educational attainment and experience, is a relatively new challenge for academics and those supporting student welfare, learning and assessment. This research showed that despite the challenging environment, student satisfaction around their teaching remained similar to previous cohorts and grades were increased.

## CONFLICT OF INTEREST

The authors have no conflicts of interest.

## Data Availability

The data that support the findings of this study are available on request from the corresponding author. The data are not publicly available due to privacy or ethical restrictions.

## References

[ahe12842-bib-0001] Astin, A. W. (1998). The changing American college student: Thirty‐year trends, 1966‐1996. The Review of Higher Education, 21(2), 115–135. https://www.muse.jhu.edu/article/30042

[ahe12842-bib-0002] Ávila, M. J. , & Rodríguez‐Restrepo, A. (2014). The importance of research in undergraduate medical education. Medwave, 14(10), e6032. 10.5867/medwave.2014.10.6032 25587714

[ahe12842-bib-0003] Bloxham, S. , & Boyd, P. (2007). Developing effective assessment in higher education: A practical guide. McGraw‐Hill Education.

[ahe12842-bib-0004] Bridge, P. , Carmichael, M. A. , Callender, J. , al‐Sammarie, F. , Manning‐Stanley, A. , Warren, M. , Gordon, C. , Drew, A. , Edgerley, J. , Hammond, M. , Hussain, Z. , Jager, C. , Mineo, R. , Pickering, V. , & Williams, C. (2018). Internationalising research methods teaching of undergraduate health professionals. Journal of Medical Imaging and Radiation Sciences, 49(1), 97–105. 10.1016/j.jmir.2017.11.003 30479296

[ahe12842-bib-0005] Brombacher‐Steiert, S. , Ehrich, R. , Schneider, C. , Müller, L. R. , Tipold, A. , & Wissing, S. (2021). Teaching clinical practical and communication skills of the clinical skills lab of the University of Veterinary Medicine Hannover, Foundation, Germany during the COVID‐19 pandemic. GMS Journal of Medical Education, 38(5), Doc86. 10.3205/zma001482 PMC8256131 34286066

[ahe12842-bib-0006] Cehrs, E. , Pelligand, L. , & Weller, R. (2020). Faculty's perception of a research project embedded in the undergraduate veterinary curriculum. Journal of Veterinary Medical Education, 47(2), 170–176. 10.3138/jvme.0318-028r1 31009275

[ahe12842-bib-0007] Deci, E. L. , & Ryan, R. M. (2000). The “what” and “why” of goal pursuits: Human needs and the self‐determination of behavior. Psychological Inquiry, 11(4), 227–268. 10.1207/S15327965PLI1104_01

[ahe12842-bib-0008] Eiszler, C. F. (2002). College Students' evaluations of teaching and grade inflation. Research in Higher Education, 43(4), 483–501. 10.1023/A:1015579817194

[ahe12842-bib-0009] Ellaway, R. , Dewhurst, D. , & Cumming, A. (2003). Managing and supporting medical education with a virtual learning environment: The Edinburgh electronic medical curriculum. Medical Teacher, 25(4), 372–380. 10.1080/0142159031000136789 12893547

[ahe12842-bib-0010] Hurst, C. S. , Baranik, L. E. , & Daniel, F. (2013). College student stressors: A review of the qualitative research. Stress and Health, 29(4), 275–285. 10.1002/smi.2465 23023893

[ahe12842-bib-0011] Iwanaga, J. , Loukas, M. , Dumont, A. S. , & Tubbs, R. S. (2021). A review of anatomy education during and after the COVID‐19 pandemic: Revisiting traditional and modern methods to achieve future innovation. Clinical Anatomy, 34(1), 108–114. 10.1002/ca.23655 32681805 PMC7404762

[ahe12842-bib-0012] Jamieson, S. (2004). Likert scales: How to (ab)use them. Medical Education, 38(12), 1217–1218. 10.1111/j.1365-2929.2004.02012.x 15566531

[ahe12842-bib-0013] Janicke, H. , Johnson, M. , Baillie, S. , Warman, S. , Stone, D. , Paparo, S. , & Debnath, N. (2020). Creating the next generation of evidence‐based veterinary practitioners and researchers: What are the options for globally diverse veterinary curricula? Journal of Veterinary Medical Education, 47, 647–658. 10.3138/jvme.2019-0098 33231517

[ahe12842-bib-0014] Karadag, E. (2021). Effect of COVID‐19 pandemic on grade inflation in higher education in Turkey. PLoS One, 16(8), e0256688. 10.1371/journal.pone.0256688 34432854 PMC8386824

[ahe12842-bib-0026] Leatherman, J. L. , & Cleveland, L. M. (2020). Student exam performance in flipped classroom sections is similar to that in active learning sections, and satisfaction with the flipped classroom hinges on attitudes toward learning from videos. Journal of Biological Education, 54(3), 328–344. 10.1080/00219266.2019.1575266

[ahe12842-bib-0015] Limniou, M. , Varga‐Atkins, T. , Hands, C. , & Elshamaa, M. (2021). Learning, student digital capabilities and academic performance over the COVID‐19 pandemic. Education Sciences, 11(7), 361. https://www.mdpi.com/2227‐7102/11/7/361

[ahe12842-bib-0016] Little, W. B. , Artemiou, E. , Conan, A. , & Sparks, C. (2018). Computer assisted learning: Assessment of the veterinary virtual anatomy education software IVALA™. Veterinary Sciences, 5(2), 58. 10.3390/vetsci5020058 PMC6024603 29921803

[ahe12842-bib-0017] Popov, S. V. , & Bernhardt, D. (2013). University competition, grading standards, and grade inflation. Economic Inquiry, 51(3), 1764–1778. 10.1111/j.1465-7295.2012.00491.x

[ahe12842-bib-0018] Routh, J. , Paramasivam, S. J. , Cockcroft, P. , Nadarajah, V. D. , & Jeevaratnam, K. (2021). Veterinary education during Covid‐19 and beyond‐challenges and mitigating approaches. Animals (Basel), 11(6), 1818. 10.3390/ani11061818 34207202 PMC8234198

[ahe12842-bib-0019] Ryff, C. D. , & Singer, B. (2003). Flourishing under fire: Resilience as a prototype of challenged thriving. In Flourishing: Positive psychology and the life well‐lived (pp. 15–36). American Psychological Association.

[ahe12842-bib-0027] Tahir, I. , Van Mierlo, V. , Radauskas, V. , Yeung, W. , Tracey, A. , & da Silva, R. (2022). Blended learning in a biology classroom: Pre‐pandemic insights for post‐pandemic instructional strategies. FEBS Open Bio., 12(7), 1286–1305. 10.1002/2211-5463.13421. PMC9249331 35488491

[ahe12842-bib-0025] Stockwell, B. R. , Stockwell, M. S. , Cennamo, M. , & Jiang, E. (2015). Blended learning improves science education. Cell, 162(5), 933–936. 10.1016/j.cell.2015.08.009 26317458

[ahe12842-bib-0020] UNESCO IESALC . (2020). COVID‐19 and higher education: Today and tomorrow. Impact analysis, policy responses and recommendations. UNESCO IESALC. https://unesdoc.unesco.org/ark:/48223/pf0000375693

[ahe12842-bib-0021] Vansteenkiste, M. , & Ryan, R. M. (2013). On psychological growth and vulnerability: Basic psychological need satisfaction and need frustration as a unifying principle. Journal of Psychotherapy Integration, 23(3), 263–280. 10.1037/a0032359

[ahe12842-bib-0022] Weinstein, N. , & Ryan, R. M. (2011). A self‐determination theory approach to understanding stress incursion and responses. Stress and Health, 27(1), 4–17. 10.1002/smi.1368

[ahe12842-bib-0023] World Health Organization . (2022). World Health Organisation Coronovirus (COVID‐19) dashboard. https://covid19.who.int/table

[ahe12842-bib-0024] Yang, C. , Chen, A. , & Chen, Y. (2021). College students' stress and health in the COVID‐19 pandemic: The role of academic workload, separation from school, and fears of contagion. PLoS One, 16(2), e0246676. 10.1371/journal.pone.0246676 33566824 PMC7875391

